# Evaluation of 24 protocols for the production of platelet-rich fibrin

**DOI:** 10.1186/s12903-020-01299-w

**Published:** 2020-11-07

**Authors:** Richard J. Miron, Jihua Chai, Masako Fujioka-Kobayashi, Anton Sculean, Yufeng Zhang

**Affiliations:** 1 The State Key Laboratory Breeding Base of Basic Science of Stomatology (Hubei-MOST) and Key Laboratory of Oral Biomedicine Ministry of Education, School and Hospital of Stomatology, Wuhan University, Wuhan, China; 2 Department of Periodontology, School of Dental Medicine, University of Bern, Bern, Switzerland; 3 Department of Cranio-Maxillofacial Surgery, Inselspital, Bern University Hospital, University of Bern, Bern, Switzerland; 4 Department of Dental Implantology, School and Hospital of Stomatology, Wuhan University, Wuhan, China

**Keywords:** Advanced platelet-rich fibrin, Leukocyte and platelet-rich fibrin, A-PRF, i-PRF, L-PRF

## Abstract

**Background:**

The aim of this study was to evaluate 24 protocols for the production of platelet rich fibrin (PRF) produced via horizontal centrifugation to better understand cell separation following protocols at various times and speeds.

**Methods:**

All protocols were compared utilizing a recent method to quantify cells in PRF in 1 mL sequential layers pipetted from the upper layer downwards until all 10 mL were harvested. In total, 960 complete blood counts (CBCs) were investigated. Both solid and liquid-based PRF protocols were investigated following 24 protocols involving 6 relative centrifugal force (RCF) values (100, 200, 400, 700, 1000 and 1200*g*) at 4 centrifugation times (3, 5, 8 and 12 min).

**Results:**

In general, platelets could more easily accumulate in the upper 4 layers when compared to leukocytes owing to their lower cellular density. Protocol time seemed to have a greater impact on the final cell layer separation when compared to the effect of speed. Protocols of greater than 8 min at 400*g* led to no leukocyte accumulation in the upper PRF layers (found specifically within the buffy coat). Protocols at or below 200*g* were unable to effectively accumulate platelets/leukocytes. The optimal centrifugation speed and time for solid-PRF ranged between 400 and 700*g* for 8 min. It was noted that variability in patient baseline platelet/leukocyte/erythrocyte counts (hematocrit) significantly affected cell layer separation. This finding was more pronounced at lower centrifugation speeds.

**Conclusions:**

Within the investigated ranges, a protocol of 700*g* for 8 min presented the highest yield of platelets/leukocytes evenly distributed throughout the upper PRF layers.

## Background

Platelet concentrates, including platelet-rich plasma (PRP) and platelet-rich fibrin (PRF), have been widely utilized in many fields of medicine as a scaffold capable of facilitating tissue regeneration [1]. PRP was first introduced over 20 years ago as a specific regenerative modality aimed at concentrating platelets from whole blood [2–6]. PRP contains an array of naturally derived autologous growth factors, including platelet-derived growth factor (PDGF), transforming growth factor beta (TGF-β), and vascular endothelial growth factor (VEGF). This set of growth factors is responsible for facilitating new blood vessel formation (angiogenesis) as well as inducing the cell migration and proliferation of various cell types [2–6]. While PRP has been widely utilized in many fields of medicine for over 2 decades, one of the major reported limitations includes its use of anticoagulants, known suppressors of clotting and thereby wound healing [2, 7, 8]. Nevertheless, the simplicity and low cost of harvesting peripheral blood and concentrating blood-derived growth factors and cells using a centrifuge has long been considered an effective and easy-to-obtain source of natural growth factors for tissue regeneration [9, 10].

PRF was thereafter developed as a second-generation platelet concentrate with the aim of anticoagulant removal [11]. This concentrate has frequently been termed leukocyte and platelet-rich fibrin (L-PRF) owing to the desired incorporation of leukocytes most commonly using a ~ 700*g* for 12-min protocol [12]. Since anticoagulants are removed, blood is subject to clotting over time within the blood collection tube, and it therefore becomes critical that the treating clinician begin centrifugation shortly following blood collection to separate blood layers [13]. One key advantage is the fibrin matrix formed in PRF, which favors the slow and gradual release of growth factors over time when compared to that in PRP [14]. Furthermore, by reducing centrifugation speeds and time, a liquid-PRF (injectable-PRF or i-PRF) was developed with an increased concentration of platelets and leukocytes [15].

Very recently, it was reported that the horizontal centrifugation of PRF was superior at accumulating platelets and leukocytes when compared to the results from standard fixed-angle centrifuges utilized to produce solid-PRF [16]. Both solid-based and liquid-based PRF matrices were obtained with up to 3.5-fold improvements in platelet/leukocyte numbers and/or concentrations [16]. While the use of horizontal centrifugation for the production of PRF remains relatively new, to date, no studies have investigated the effect of various centrifugation protocols on the final outcomes of PRF. Therefore, the aim of the present study was to optimize both solid- and liquid-based PRF matrices on a horizontal centrifuge by investigating a series of 24 protocols. The following 6 RCF values (100*g*, 200*g*, 400*g*, 700*g*, 1000*g* and 1200*g*) were investigated and compared at 4 different centrifugation times (3, 5, 8 and 12 min) to determine cell numbers and cellular concentrations.

## Methods

### Preparation of PRF

Blood samples were collected with informed consent from 4 volunteer donors. All procedures involving human participants in this study were performed in accordance with the ethical standards of the institutional and/or national research committee and with the 1964 Declaration of Helsinki and its later amendments. All blood samples were collected in Wuhan, China and utilized in accordance to the guidelines by The University of Wuhan ethical standards and guidelines via an IRB process (Internal Review Board, University of Wuhan). An ethical request was waived by the IRB for this study since blood was not used as identifiable sources [15]. Blood samples were collected with the written informed consent of 4 volunteer donors.

The factors that affect fibrin clot formation and structure include genetic factors, acquired factors (such as abnormal concentration of thrombin and factor XIII in plasma, blood flow, platelet activation, oxidative stress, hyperglycemia, hyperhomocysteinemia, medications, and cigarette smoking), and other parameters (such as microgravity, pH, temperature, reducing agents, and concentration of chloride and calcium ions) [17]. All donors were confirmed to be without any of the above conditions. The CBC of the donors were also investigated prior to beginning the experiments to confirm standard cell count ranges.

**Fig. 1:**
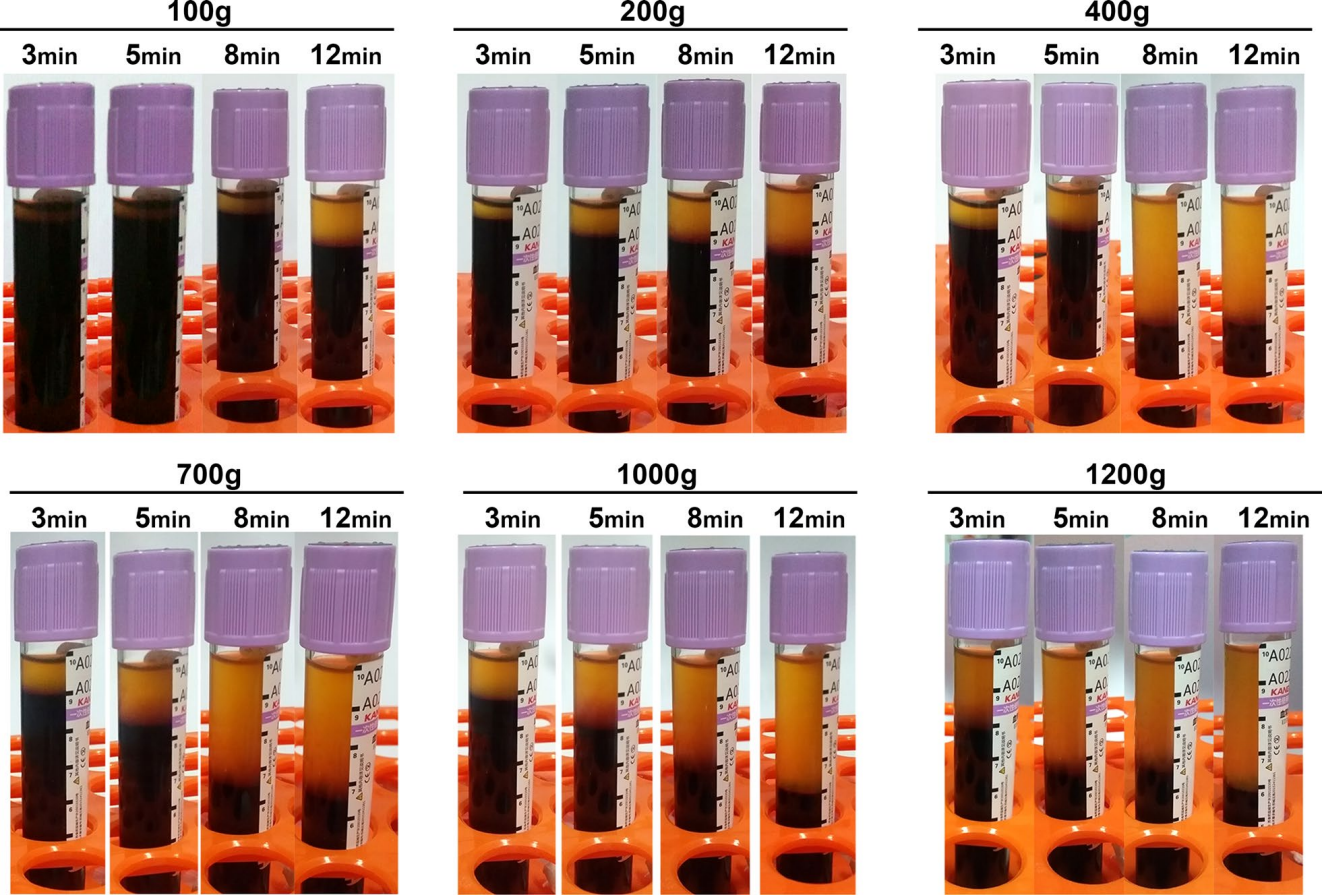
Clinical image demonstrating the plasma layer separation for the 24 protocols investigated in this study. Note that while some protocols reveal roughly identical plasma layer separation, the underlying cellular content in the various protocols may be drastically different

The production of PRF was obtained utilizing an Eppendorf 5702 horizontal centrifuge (Hamburg, Germany). Each of the 4 volunteers donated 24 vials of blood in standard 10 mL plastic collection tubes (240 mL total; Chixin Biotech, Wuhan, China). The following 24 protocols were investigated, which included 6 different RCF values of 100*g*, 200*g*, 400*g*, 700*g*, 1000*g* and 1200*g* and 4 centrifugation times of 3, 5, 8 and 12 min, according to Fig. 1.

### Quantification of cells found in PRF

**Fig. 2:**
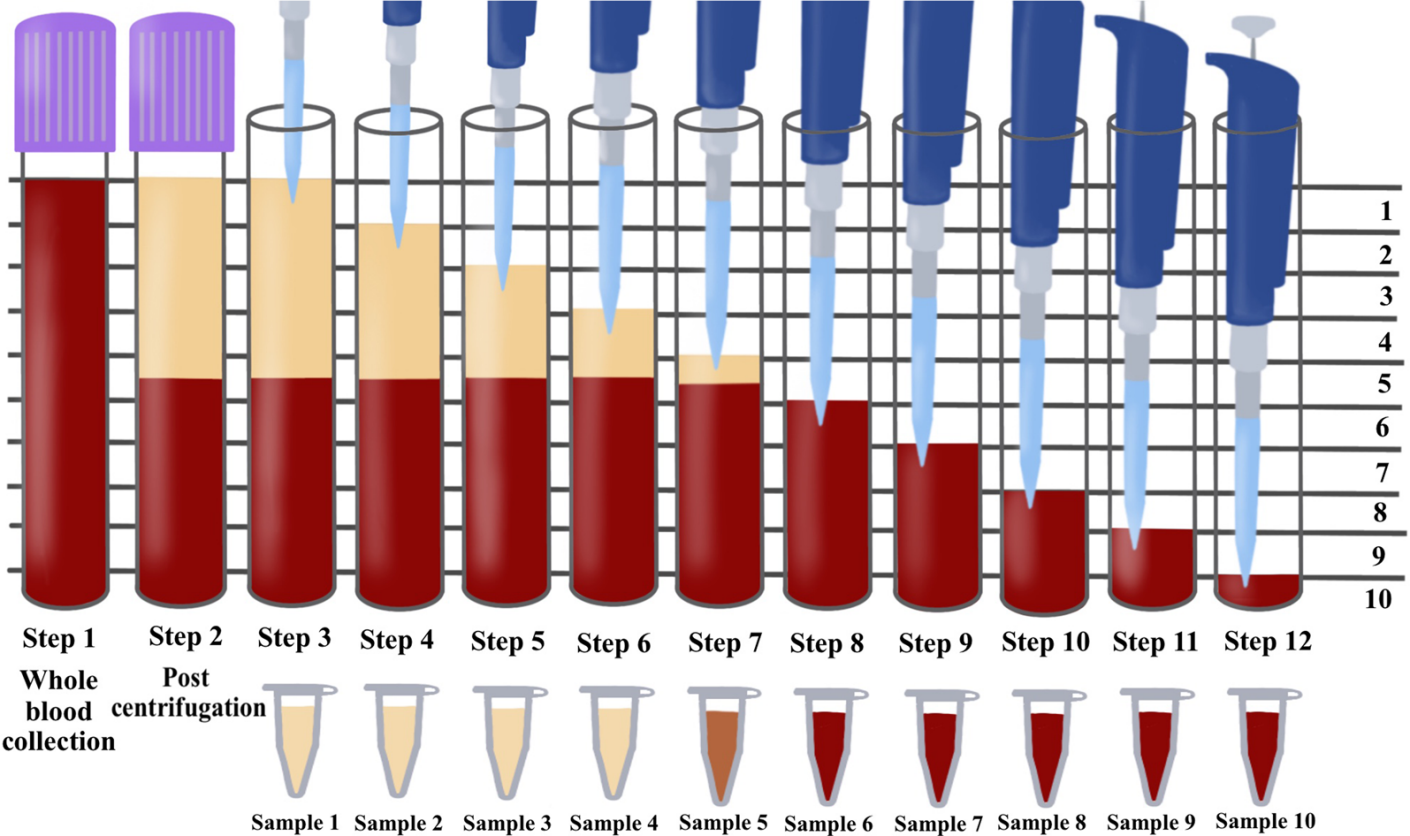
Illustration demonstrating the proposed novel method to quantify cell types following the centrifugation of PRF. One of the limitations of the current methods utilized to investigate PRF cellular content from the whole plasma layer is the inability to accurately determine where cells migrate following centrifugation. By utilizing the proposed technique using the sequentially pipetting 1 mL layers from the top layer downwards, it is possible to quantify cell numbers in 10 samples from CBC analysis and accurately determine the precise location of each cell layer following centrifugation at various protocols. Note that one layer (in this case, layer 5) will contain both yellow plasma and red blood cells. This effect is figuratively depicted with arrows to demonstrate the location of the buffy coat where a higher concentration of platelets/leukocytes is typically found. Reprinted with permission [16]

**Fig. 3:**
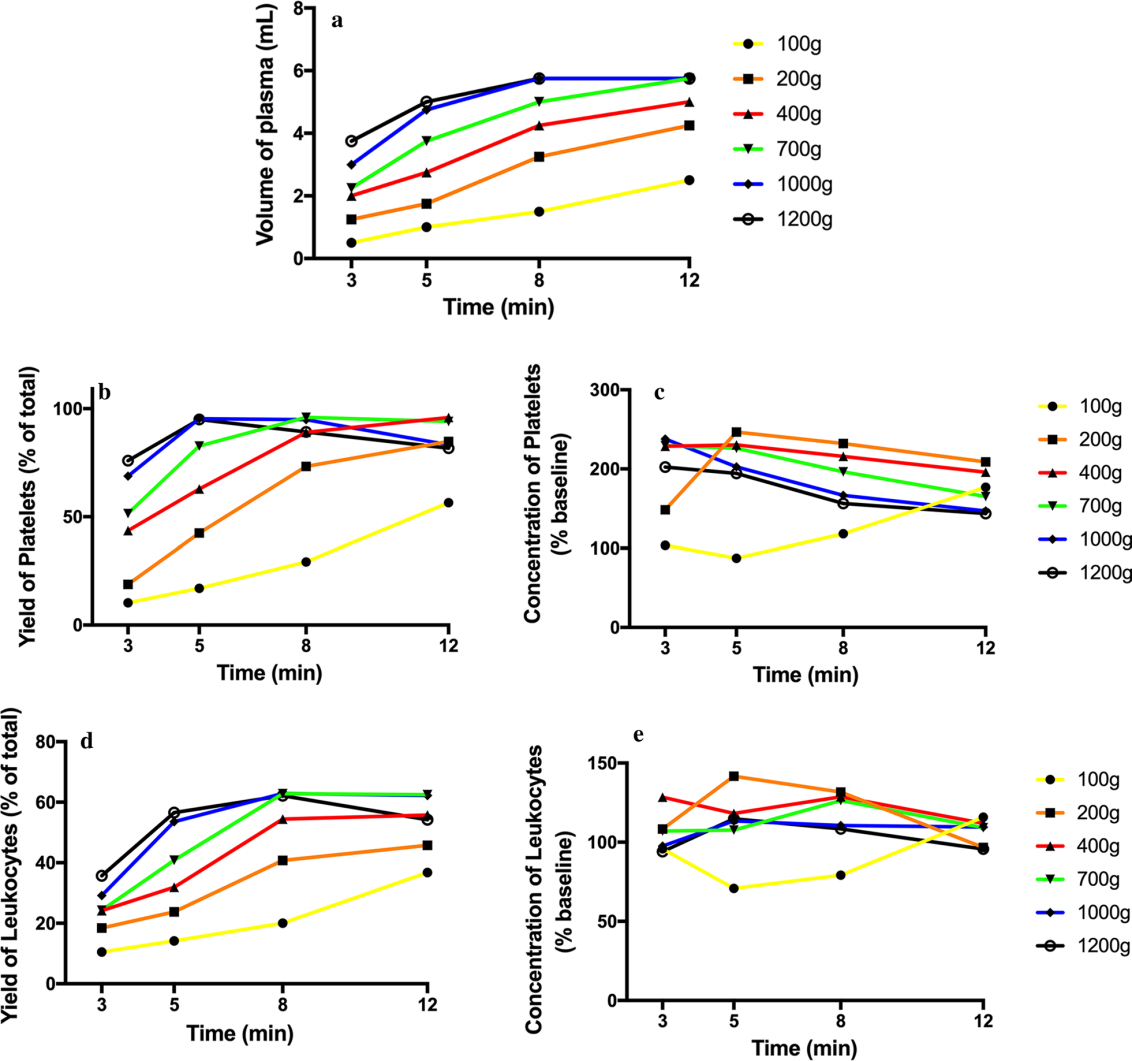
Evaluation of 24 protocols utilized for the production of PRF. Data includes final volume (mL), total leukocyte and platelet yields (% of the total from 10 mL) as well as concentration of leukocytes and platelets above baseline values (% increase)

**Fig. 4:**
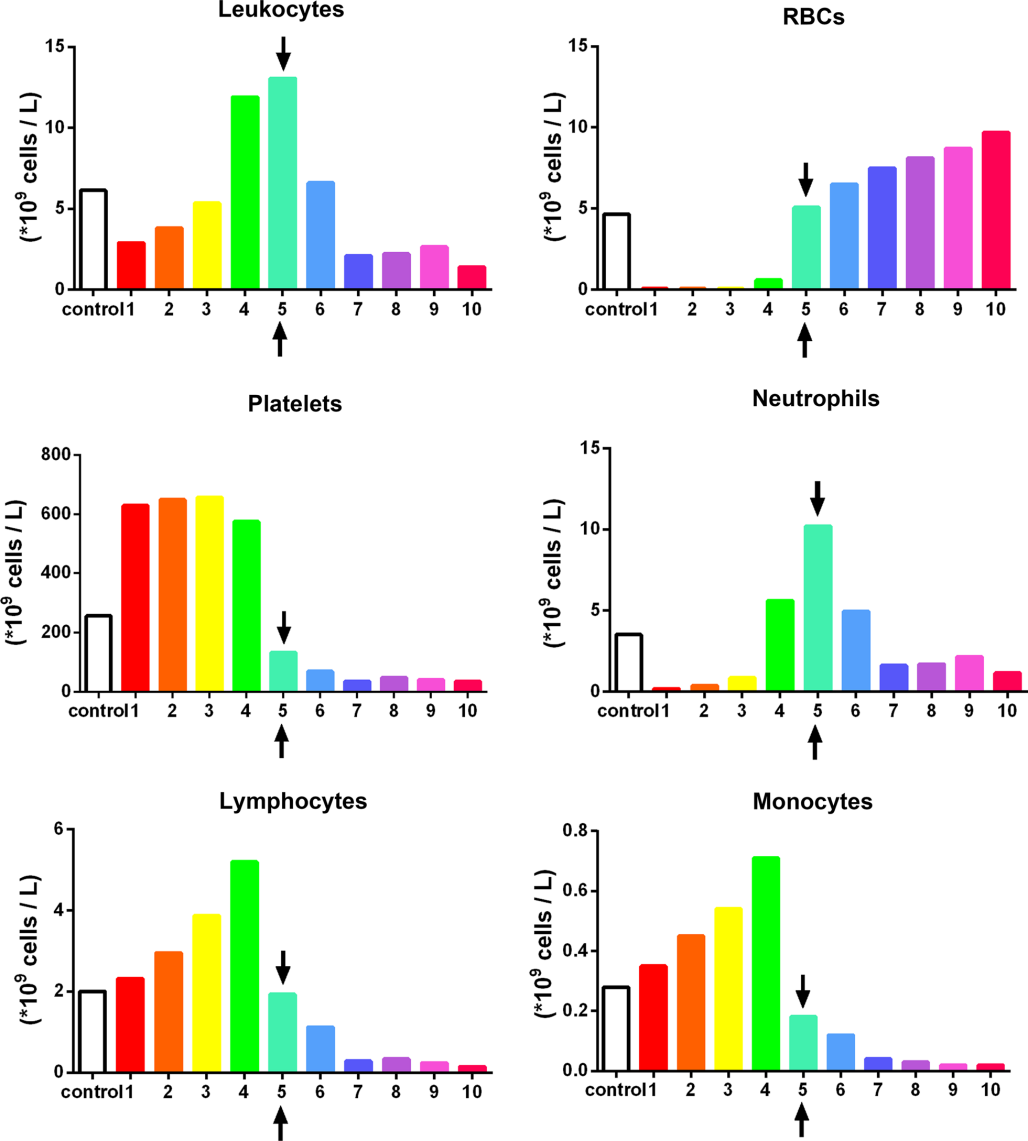
The concentration of cell types in each layer from 1 mL down to the 10th 1-mL sample utilizing the solid-PRF horizontal centrifugation protocol (700*g* for 8 min). Notice that most of the platelets are evenly distributed throughout the upper plasma layer. Similarly, white blood cells are primarily distributed throughout the upper 4 layers, though not as even as platelets owing to their slightly higher cellular density (Arrows represent the separation between the plasma and red blood cell layer (buffy coat)). Reprinted with permission [16]

**Fig. 5:**
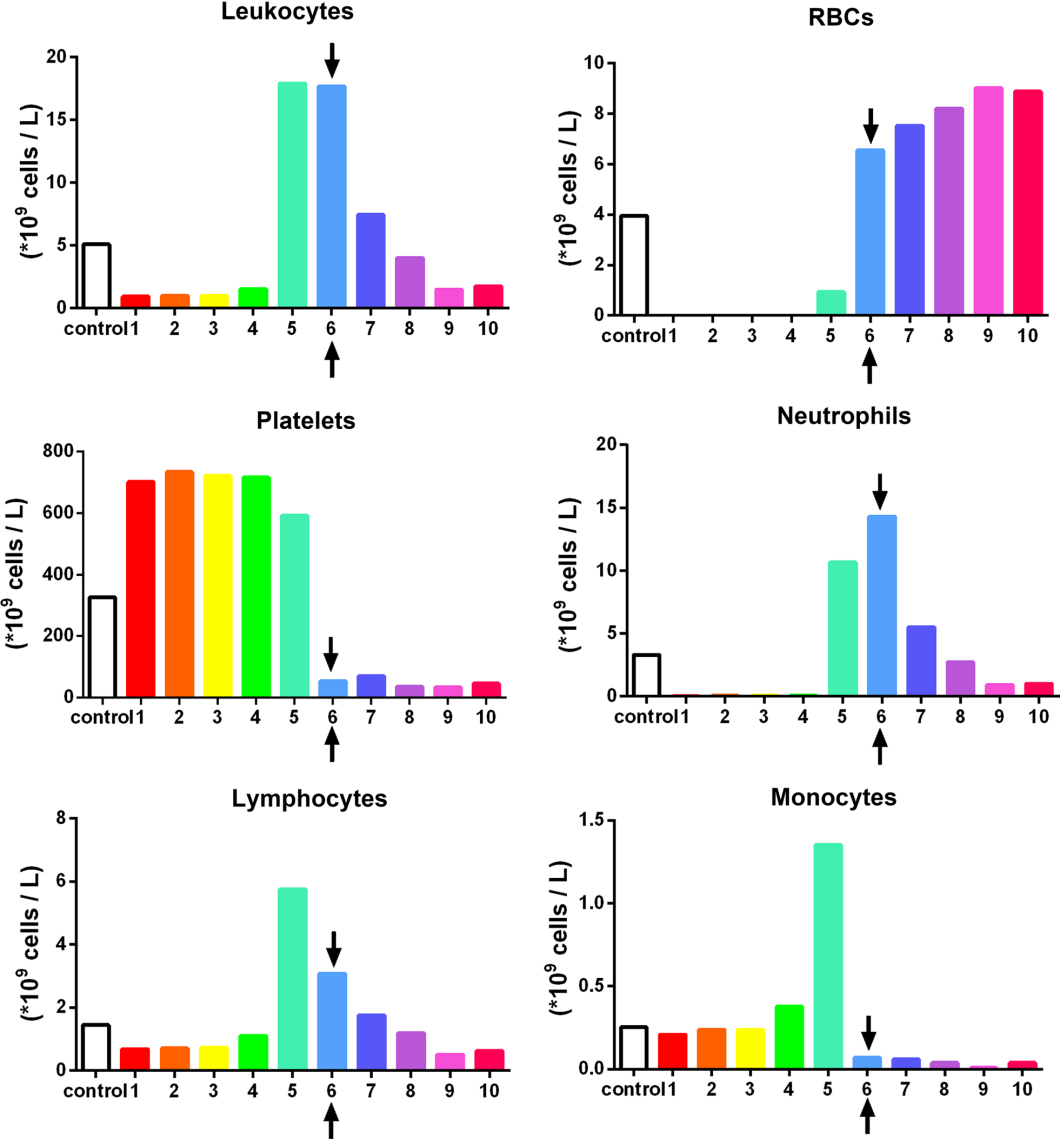
Observed variability of the concentration of cell types in each layer from 1 mL down to the 10th 1-mL sample utilizing the same solid-PRF horizontal centrifugation protocol (700*g* for 8 min) as depicted in Fig. 4 (owing to difference in patient gender and age). Notice that when compared to Fig. 4, more leukocytes are found within the buffy coat layer. This finding is a result of a patient demonstrating lower hematocrit counts (typically occurring in older female patients), thereby resulting in easier cell-layer separation and more pronounced accumulation within the buffy coat layer (arrows represent the separation between the plasma and red blood cell layer (buffy coat))

**Fig. 6:**
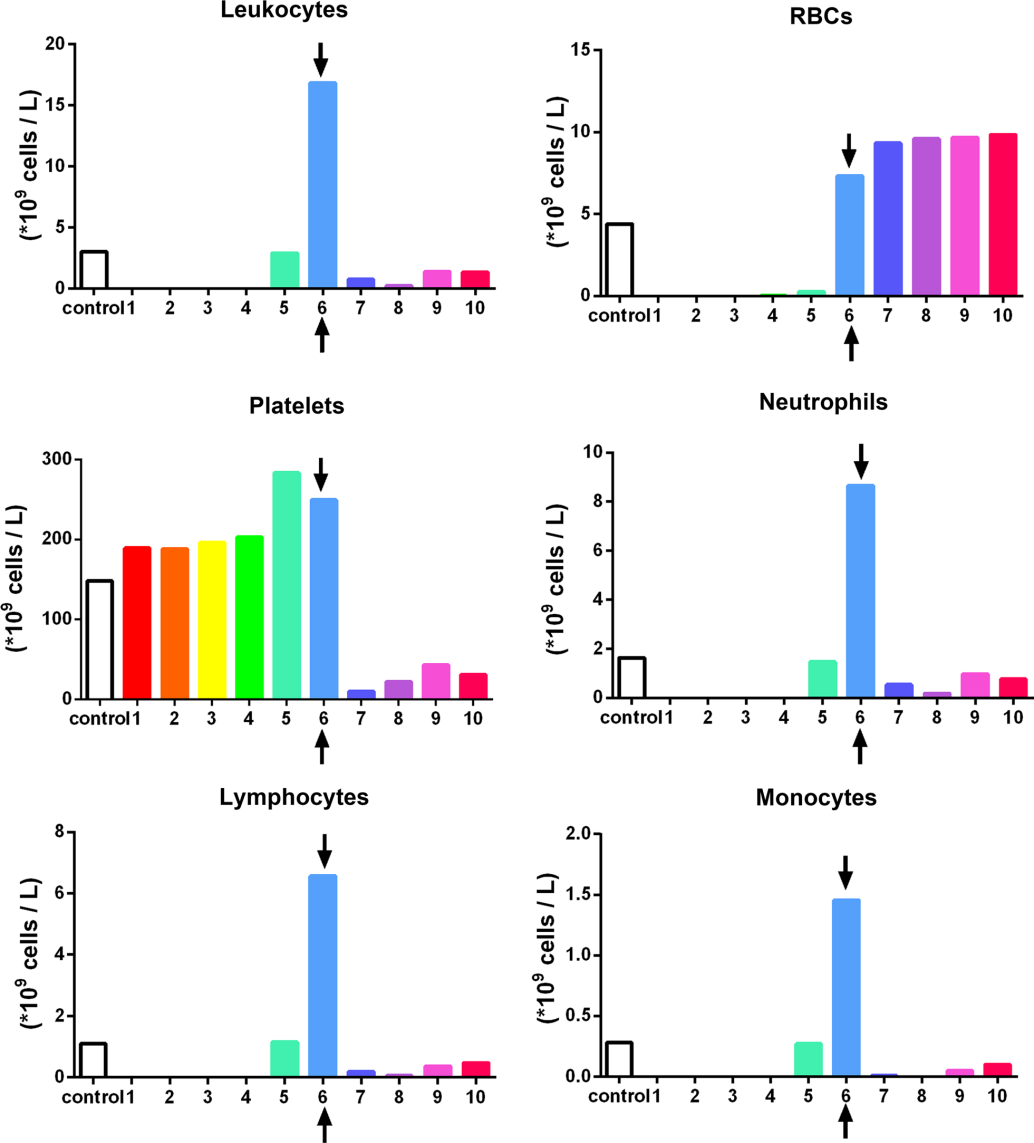
The concentration of cell types in each layer from 1 mL down to the 10th 1-mL sample utilizing a solid-PRF horizontal centrifugation protocol at 700*g* for 12 min. Notice that when compared to Fig. 4, the higher centrifuge time resulted in more leukocytes concentrated within the buffy coat layer. This finding demonstrates that over time, cells migrate more toward the buffy coat layer with extended periods of time or extended RCF values (Arrows represent the separation between the plasma and red blood cell layer (buffy coat))

To accurately quantify the cells found in PRF, a sequential pipetting protocol was utilized as previously described [16]. The protocol involves sequentially pipetting 1 mL layers from the top layer down to the bottom 1 mL layer, as depicted in Fig. 2. Notably, during the harvesting of the 1 mL layers, one sample was collected between the plasma/buffy coat and the red blood cell (RBC) layer. This layer was marked within each figure to represent the location of the buffy coat and displayed graphically with arrows (see Figs. 3, 4, 5 and 6 in Results as an example) to represent the separation between the yellow plasma and RBC layers. Furthermore, the total volumes (in mL) of plasma were recorded from each protocol to calculate the total cell numbers and concentrations following each protocol. Each blood sample (1 mL) was then then placed into 1.5 mL plastic tubes with anti-coagulants (2 mg/mL EDTA-K2) to allow for the blood samples to thereafter be sent for complete blood count (CBC) analysis, in which the total number of leukocytes, RBCs platelets, neutrophils, lymphocytes and monocytes were calculated from each sample using a Sysmex XN-550 (Sysmex Corporation, Kobe, Japan) based on fluorescence flow cytometry. In total, 960 samples were sent for CBC analysis and investigated accordingly thereafter. Each sample was displayed graphically using GraphPad Prism 6.0 software (GraphPad Software, Inc., La Jolla, CA, USA). Data averages with standard errors were presented in terms of percentages of total yield of platelets and leukocytes as well as final concentration from baseline values. Furthermore, informative trends were also reported from the data presented.

## Results

### Cell numbers following centrifugation using various protocols

Following centrifugation utilizing the various protocols, 1 mL sequential layers were sent for CBC analysis, and each protocol incorporated 10 CBCs from each donor sample when compared to baseline levels. Several trends were observed within the present study owing to the extensive number of CBC evaluations (960 total evaluations) performed throughout the study.

Figure 3 demonstrates the overall final volume of plasma of each of the protocols, along with the total yield and concentrations of platelets and leukocytes above baseline values. Note that of each of the protocols, a 200*g* for 5 min protocol led to the highest concentration of platelets/leukocytes. The best yield of leukocytes was achieved after centrifugation for 8 min utilizing the 700, 1000 and 1200*g* protocols. Of those the highest concentration was achieved at 700*g* for 8 min (owing to the reduced total plasma volume). Another noteworthy trend that was apparent was that as centrifugation time was increased, a general increase in percent yield was observed, however generally speaking, an overall decrease in concentration was observed (Fig. 3). Furthermore, it was apparent that certain centrifugation protocols that were too reduced in RCF (such as 100*g*) typically did not lead to adequate yield of cells. Furthermore, protocols that were too elevated led to a reduction in yield and/or concentrations.

For instance, a significantly elevated protocol (1000*g* or more for 5 min) or a lengthier 12-min protocol at 400*g* or more was either too high or lengthy to adequately distribute platelets/leukocytes within the upper plasma layers. For the production of even distribution throughout the upper plasma layers of PRF, the protocol best distributing platelets and leukocytes within the upper 3–5 mL plasma layer was observed at 700*g* for 8 min (Fig. 4). Note that platelets were evenly distributed in the upper PRF layer, whereas leukocytes, though primarily accumulated within the upper layers, were shifted slightly more toward the buffy coat owing to their slightly higher cellular density (Fig. 4). Their overall yield was reduced in comparison to platelets (Fig. 3). Interestingly, when patients displayed slightly lower total RBCs (less hematocrit – typically in women and elderly populations), cell separation occurred slightly faster. This effect results in WBCs typically found more closely related to the buffy coat layer (Fig. 5). Similarly, when the protocols were extended past 8 min (12 min), the cells continued to accumulate within the buffy coat layer as opposed to being evenly distributed throughout the PRF clot (Fig. 6).

**Fig. 7:**
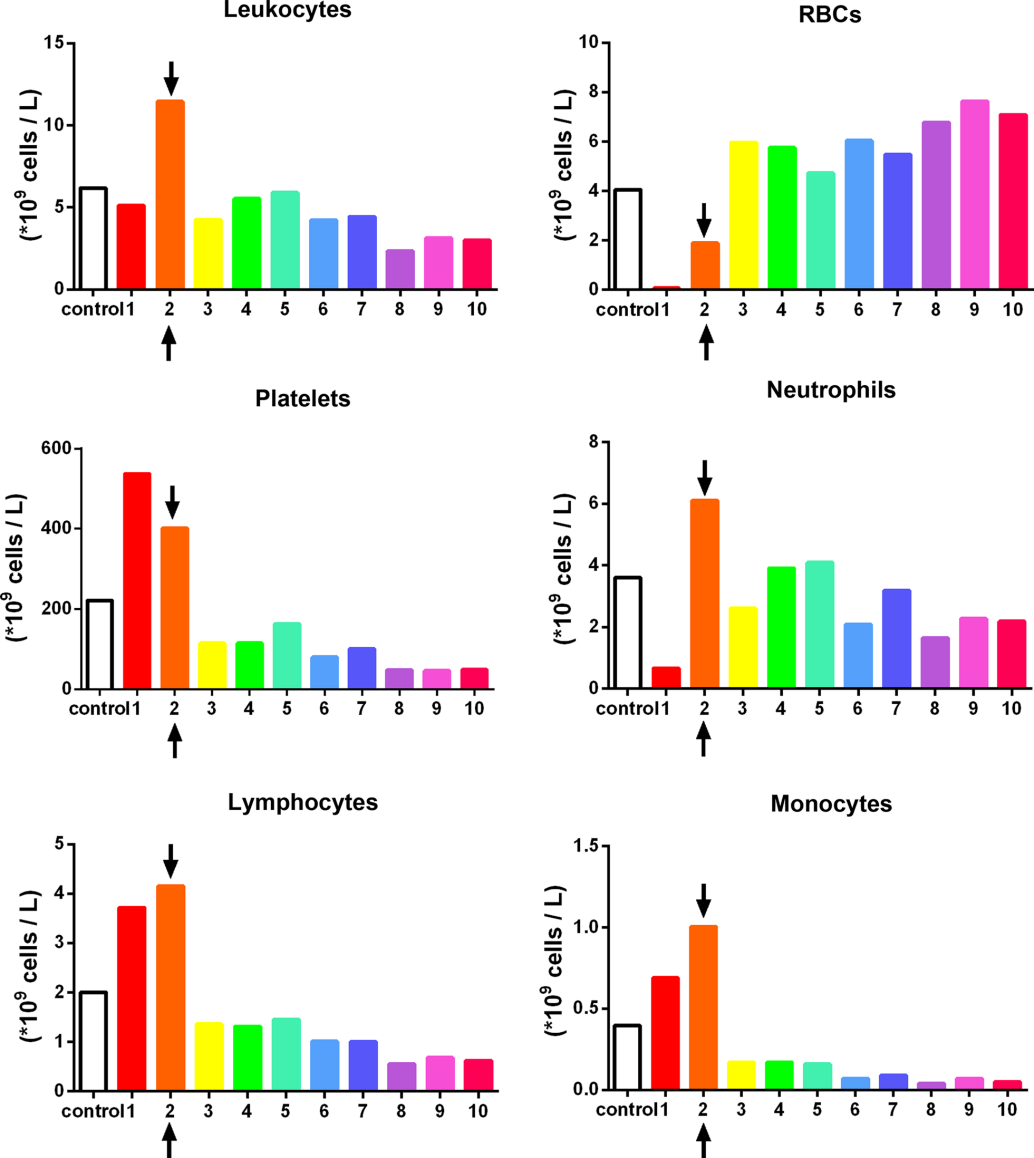
The concentration of cell types found in each layer from 1 mL down to the 10th 1-mL sample utilizing the liquid-PRF protocol of 200*g* for 5 min. Notice that while not as many cells are located in the plasma layer, a higher concentration of platelets and leukocytes can be found owing to the reduced plasma volume when compared to that in the solid-PRF protocols demonstrated in Fig. 3 (Arrows represent the separation between the plasma and red blood cell layer (buffy coat))

In contrast, PRF utilized in a clinical setting as a liquid formulation (injectable) does not necessarily aim to accumulate the highest possible yield of total platelets/leukocytes in the upper plasma layer but instead accumulates the highest concentration of platelets and leukocytes for small volume injections/mixing with biomaterials. As such, a shorter and slower centrifugation protocol is utilized to concentrate the cells in a smaller plasma layer (by volume). In the present study, protocols at 100*g* were inadequately effective at separating cell layers, and even a 200*g* protocol for 3 min did not result in sufficient concentrations of platelets/leukocytes. The protocol which demonstrated the highest concentration of platelets and leukocytes was observed at 200*g* for 5 min (Fig. 3; cell distribution depicted in Fig. 7). Note that while the total yield of platelets and leukocytes was lower in the 200*g* for 5 min protocol when compared to 700*g* for 8 min, the overall concentration was higher (Table 1).

**Table 1:** Evaluation of 24 protocols utilized for the production of PRF

	100*g*	200*g*	400*g*	700*g*	1000*g*	1200*g*
*Final volume (mL)*						
3 min	0.5 ± 0.29	1.25 ± 0.48	2.13 ± 0.31	2.25 ± 0.25	3.00 ± 0.41	3.75 ± 0.25
5 min	1 ± 0.58	1.75 ± 0.25	2.75 ± 0.25	3.75 ± 0.48	4.75 ± 0.25	5.00 ± 0.41
8 min	1.5 ± 0.65	3.25 ± 0.48	4.25 ± 0.48	5.00 ± 0.41	5.75 ± 0.25	5.75 ± 0.25
12 min	2.5 ± 0.96	4.25 ± 0.48	5.00 ± 0.41	5.75 ± 0.25	5.75 ± 0.25	5.75 ± 0.25
*Total leukocytes (% total of 10 mL)*						
3 min	10.5 ± 0.71	18.42 ± 5.01	24.17 ± 3.08	24.33 ± 4.30	29.17 ± 5.11	35.67 ± 4.38
5 min	14.17 ± 1.18	23.75 ± 2.67	31.92 ± 3.10	40.83 ± 8.75	53.58 ± 3.48	56.58 ± 3.33
8 min	20 ± 7.07	40.79 ± 2.46	54.42 ± 5.63	62.83 ± 6.11	*62.92* ± *2.75*	62.17 ± 6.13
12 min	36.75 ± 4.76	45.75 ± 2.47	55.75 ± 4.25	62.50 ± 4.98	62.25 ± 3.45	54.25 ± 4.57
*Concentration of leukocytes (% baseline)*						
3 min	95.83 ± 5.89	108.33 ± 6.31	128.47 ± 12.52	107.08 ± 12.52	97.57 ± 10.40	94.03 ± 6.59
5 min	70.83 ± 5.89	*141.67* ± *17.01*	118.19 ± 12.90	107.64 ± 12.90	113.42 ± 7.59	114.76 ± 9.04
8 min	79.17 ± 5.89	131.67 ± 14.06	128.63 ± 3.00	125.25 ± 9.09	110.56 ± 9.37	108.47 ± 10.36
12 min	115.83 ± 4.12	96.71 ± 4.82	111.88 ± 3.37	109.17 ± 8.94	109.58 ± 10.83	95.69 ± 11.79
*Total platelets (% total of 10 mL)*						
3 min	10.35 ± 1.38	18.81 ± 3.04	43.63 ± 6.13	51.47 ± 5.24	68.75 ± 6.13	75.91 ± 4.91
5 min	16.98 ± 1.92	42.49 ± 5.68	62.85 ± 5.61	82.76 ± 5.84	95.37 ± 1.61	94.98 ± 1.39
8 min	29.17 ± 5.89	73.32 ± 7.82	89.03 ± 3.61	*96.06* ± *2.33*	94.92 ± 3.26	89.21 ± 1.64
12 min	56.52 ± 0.92	84.88 ± 3.53	95.94 ± 1.71	94.09 ± 3.49	83.29 ± 6.89	81.69 ± 3.78
*Concentration of platelets (% baseline)*						
3 min	103.54 ± 13.85	148.37 ± 18.84	228.55 ± 17.33	229.56 ± 7.03	237.83 ± 19.39	202.37 ± 14.43
5 min	87.40 ± 13.11	*246.53* ± *15.47*	230.32 ± 13.75	226.07 ± 17.03	202.67 ± 12.41	194.31 ± 17.79
8 min	118.06 ± 9.82	232.06 ± 17.75	215.84 ± 19.59	196.29 ± 18.40	166.54 ± 12.08	156.45 ± 10.10
12 min	176.82 ± 2.30	208.86 ± 17.64	195.88 ± 17.23	165.15 ± 12.57	146.76 ± 17.40	143.53 ± 12.30

Data includes final volume (mL), total leukocyte and platelet yields (% of the total from 10 mL) as well as concentration of leukocytes and platelets above baseline values (% increase) (data represents average ± standard error)

One interesting observation in the present study was the variability observed among various donors when centrifugation was performed with identical protocols in different aged/gender populations (Additional file 1: Figs. S1–S3). Note the difference in cell layer separation among 3 donor samples centrifuged at 700*g* for 5 min. While in donor 1, the plasma layer separation occurred in layer 3 with ~ 3 mL of plasma (Additional file 1: Fig. S1), donor 3 demonstrated plasma layer separation in layer 5 with ~ 5 mL of plasma with the exact same protocol (Additional file 1: Fig. S3). This finding demonstrates a more than 50% increase in the total amount of plasma when following identical protocols owing to variability among donors. This result was due to differences in the initial baseline values of the platelets, leukocytes and RBCs (hematocrit levels) and may represent additional variability among a standard population.

## Discussion

PRF has gained tremendous momentum in recent years as a natural concentration of autologous growth factors capable of stimulating tissue regeneration. Despite its widespread use, to date, very little scientific data exist from studies investigating centrifugation protocols. In 2014, lower centrifugation speeds were proposed as a means to better accumulate growth factors and cells within the upper platelet-rich layers by modifying L-PRF protocols from 700*g* down to 200*g* [18]. An approximately 20% increase in platelet concentration could be observed following these lower speed centrifugation protocols [16]. While these previous methods based on histological observations allow for a relative estimate of the cells found in the various blood cell layers, the new methodology recently proposed by our group [16] allows for the precise quantification and concentration of cells in 1 mL incremental layers following centrifugation. This protocol provides researchers with a better ability to understand the events occurring following centrifugation at various protocols.

The aim of the present study was therefore to utilize the previously established method to investigate 24 protocols on a horizontal centrifuge. Table 2 demonstrates the properties of blood cells, including their density, frequency, surface areas and surface volumes. Note that while platelets are the least dense of the group, minor differences occur between leukocytes and RBCs, which greatly impacts the ability to separate blood cell layers based on density. Furthermore, while white blood cells (WBCs) are less dense when compared to RBCs (1055–1085 kg/m^3^ vs. 1095–1100 kg/m^3^), note that WBCs are generally larger in size when compared to RBCs (surface area 330 vs 140 µm^2^, radium 5–7.5 vs. 4 µm and volume of 200 vs. 92 µm^3^). In addition, RBCs greatly outnumber WBCs by over 1000-fold (5 million RBCs vs 5000 WBCs per µL). These findings greatly influence the ability to separate cell types in a centrifuge based on density.

**Table 2:** Properties of the blood cells, including shape, density, surface area, radius, and frequency distribution in whole blood

	Platelets	WBC	RBC
Density (kg/m^3^)	1040–1065	1055–1085	1095–1100
Frequency (1/µL)	200,000	5000	5,000,000
Surface (µm^2^)	28	330	140
Radius (µm)	11.5	5–7.5	4
Volume (µm^3^)	14	200	92
Shape	Irregular disk	Spherical	Biconcave

**Fig. 8:**
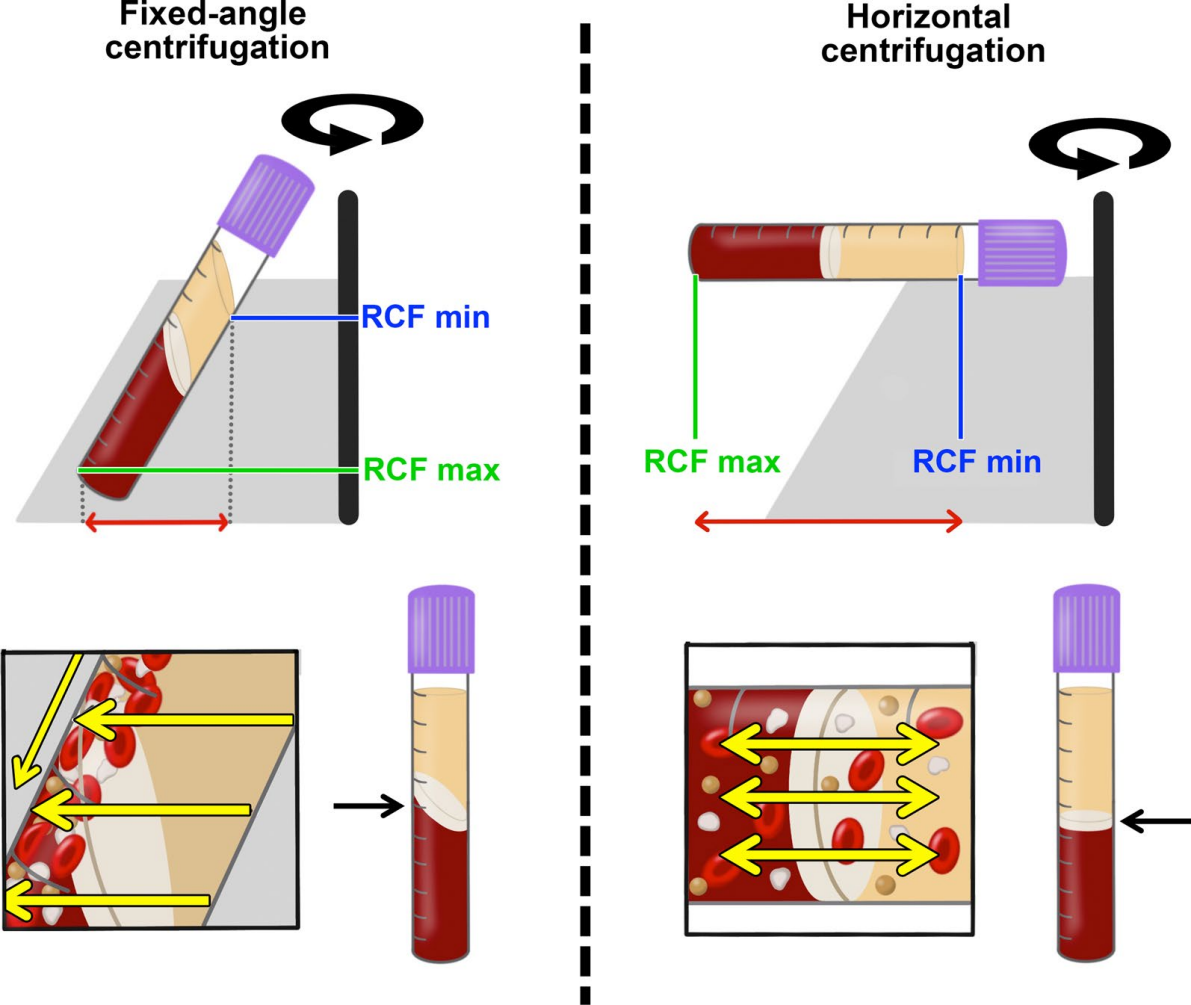
Illustrations comparing fixed-angle and horizontal centrifuges. With horizontal centrifugation, a greater separation of blood layers based on density is achieved owing to the greater difference in RCF-min and RCF-max. Following centrifugation on fixed-angle centrifuges, blood layers do not separate evenly, and as a result, an angled blood separation is observed. In contrast, horizontal centrifugation produces even separation. Owing to the large RCF values (~ 200–700*g*), the cells are pushed toward the outside and downwards. On a fixed-angle centrifuge, cells are pushed toward the back of centrifugation tubes and then downwards/upwards based on cell density. These g-forces produce additional shear stress on cells as they separate based on density along the back walls of centrifugation tubes. In contrast, horizontal centrifugation allows for the free movement of cells to separate into their appropriate layers based on density, allowing for better cell separation as well as less trauma/shear stress on cells. Modified from Miron et al. [16].

A horizontal centrifuge was utilized in this study owing to its better ability to separate blood cell layers (up to a 4 times greater yield in platelets/leukocytes) and avoid the grouping of cells along the back distal walls of centrifugation tubes as observed on fixed angle-centrifuges (Fig. 8) [16]. Two advantages were previously noted utilizing horizontal centrifugation [16]. First, a completely horizontal position produced from a swing-out bucket allows for the greatest differential between the minimum and maximum radius found within a centrifugation tube (Fig. 8). This effect allows for a greater ability to separate cell layers based on disparities between the RCF-min and RCF-max produced within a tube. Second, a fixed-angle centrifuge results in more trauma to cells along the back walls of centrifugation tubes [16]. A recent study demonstrated that fixed-angle centrifugation led to uneven concentrations of cells and growth factors, with the majority of cells found on the back distal side of PRF tubes with uneven concentrations found throughout PRF clots [19]. That paper highlights yet another effective method whereby cell layer separation can be evaluated histologically via sectioning of clotted PRF membranes layer by layer [19]. Once allocated along the back distal tube surfaces, difficulty exists in effectively separating blood cells leading to lower yields of cells [16]. For these reasons, horizontal centrifugation was recently proposed as a means to better separate blood cell layers for the production of PRF when compared to the results from fixed-angle centrifuges commonly utilized to produce solid-PRF protocols [16].

In general, platelets were evenly distributed throughout a variety of protocols within the upper 3–6 plasma-rich layers (Figs. 3, 4, 5, 6, 7). Additionally, white blood cells required more pristine fine-tuning to reach adequate harmony within the upper plasma layers. Protocols within the 400–700*g* (5–8 min) range were able to accumulate platelets more evenly distributed throughout the upper layers, whereas slightly better optimization was required to effectively spread leukocytes within the upper plasma layers. Note that even though platelets were evenly distributed throughout the upper plasma layer, white blood cells typically did not fully distribute evenly throughout the upper 4–6 layers, with the greatest concentration remaining at the buffy coat layer (arrows, Fig. 4).

The most surprising finding from the present study was the marked difference in cell layer separation that occurred at the various protocols. Notably, inadequate testing of protocols may drastically lead to a poor yield of cells evenly distributed throughout the PRF clot. A 400–700*g* protocol for 8 min was required to effectively separate the cell layers for solid-PRF protocols. Interestingly, there was also variability reported between patients. As erythrocytes and other blood components also play a role in coagulation, as well as the hematocrit counts vary between patients and affect the size of PRF membranes [20], the present study also showed that not only the total plasma volume but also the cellular content of the layers were altered (Additional file 1: Figs. S1–S3). We have previously shown that both females and patients above the age of 65 typically have larger membranes as a result of their lower hematocrit counts [20]. Future research is needed to better understand how centrifugation speed and time may be further optimized to result in similar final cell concentrations from patients with different starting blood cell counts. In summary, patients with lower cell blood counts would theoretically require lower centrifugation speeds, and further computational research is likely needed to optimize the protocols.

Currently, one standard in the field of PRF is the novel use of injectable i-PRF [15]. Previously, our research group found that only slight increases in platelets and leukocytes were noted with failure to adequately accumulate cells in the upper plasma layer owing to extremely low RCF values (60*g*) and centrifugation times (3–4 min) [16]. While our group reported a 33% increase in cell concentrations in i-PRF protocols, other groups have more recently reported similar findings [21]. In a study titled “Injectable platelet-rich fibrin: cell content, morphological, and protein characterization”, only a slight increase in platelets (less than 33%) and leukocytes was observed following i-PRF protocols, with decreases in VEGF reported when compared to that in whole blood [21]. Altogether, these studies confirm that previously utilized i-PRF protocols (~ 60*g* for 3–4 min on a fixed-angle centrifuge) are inadequately effective at separating blood cell layers owing to their considerable reduction in centrifugation speed and time. We found in the present study, that protocols at 100*g* or lower were inefficient at accumulate platelets and leukocytes in the upper plasma layer. It must therefore be highlighted that a limit exists with respect to the ‘low-speed centrifugation concept’, with further research being needed to further optimize fixed-angle centrifugation systems.

In the present study, the use of PRF produced via horizontal centrifugation with the highest concentration of platelets and leukocytes was observed at higher and longer centrifugation speeds and times. By combining an increase in speed and time, up to a fourfold increase in platelet/leukocyte concentration and/or yield was observed when compared to the results of previously utilized i-PRF protocols produced on a fixed-angle centrifuge [16]. The present study further questions the use of trademarks such as ‘leukocytes and platelet-rich fibrin’ or ‘L-PRF’. As observed in our study, changes in centrifugation speeds and times can alter the final leukocyte concentrations/yields and may even further lead to actual reductions in leukocyte number when compared to that in whole blood (Table 1). Previously, we have shown how the production of L-PRF on a fixed angle-centrifuge actually led to an actual decrease in final leukocyte concentrations when compared to those in whole blood [16]. In the present study, a protocol of 400–700*g* for 8 min produced via horizontal centrifugation led to increases in leukocyte concentrations. To the best of the authors knowledge, these procedures are the only protocols established with a reported increase in leukocyte concentrations found in solid-PRF-based protocols.

One of the principal advantages of utilizing the present methodology to investigate cell layer separations was the ability to locate and quantify cell layer changes accurately over time and at different RCF values. The ability to quantify 960 CBCs favored a better understanding of the cell layer separation of PRF-based protocols. Future research aims to better understand how time versus speed affect blood cell layer changes and how patient variability can be fine-tuned to further optimize the centrifugation protocols for PRF in patients with different hematocrit levels. This study further highlights the need for additional research in the field to further improve the understanding of centrifugation protocols for the production of PRF.

## Conclusion

The present study found that centrifugation speed and time greatly influenced the final cell counts in the upper PRF layers. In general, when compared to leukocytes, platelets more easily separated evenly into the plasma layers owing to their lower cellular density. Protocol time seemed to have a greater impact on the final cell layer separation when compared to speed. The optimal centrifugation speed and time ranged between 400 and 700*g* for 8 min for solid-PRF protocols (greater yield with evenly distributed cells) and 200–400*g* for 5 min for liquid-PRF (highest concentration of platelets/leukocytes). Notably, variability in patient baseline platelet/leukocyte counts significantly affected cell layer separation. This finding was more pronounced at lower centrifugation speeds.

## Supplementary information

**Additional file 1**. Variability of cell layer separation following the same protocol in 3 different individuals. One interesting observation in the present study was the variability observed among various donors when centrifugation took place at identical protocols. Note the difference in cell layer separation among 3 patients centrifuged at 700*g* for 5 min. While in donor one, the plasma layer separation occurred in layer 3 (**Figure S1**), donor 3 demonstrated separation in layer 5 (**Figure S2**) and demonstrated a greater than 50% increase in the total amount of plasma following identical protocols. The majority of donor samples tended to show separation at layer 4, as depicted in **Figure S3**. (Arrows represent the separation between the plasma and red blood cell layer (buffy coat)).

## Data Availability

The datasets used and/or analyzed during the current study available from the corresponding author on reasonable request.

## Abbreviations

CBC: Complete blood count
i-PRF: Injectable platelet rich fibrin
L-PRF: Leukocyte and platelet rich fibrin
PDGF: Platelet derived growth factor
PRF: Platelet rich fibrin
PRP: Platelet rich plasma
RBC: Red blood cell
RCF: Relative centrifugal force
TGF-β: Transforming growth factor-beta
VEGF: Vascular endothelial growth factor
WBC: White blood cell
