# Supplementary figures and images for: Evaluation of 24 protocols for the production of platelet-rich fibrin
Source: BMC Oral Health. 2020 Nov 7;20:310. doi: 10.1186/s12903-020-01299-w (PMC7648315; doi:10.1186/s12903-020-01299-w)

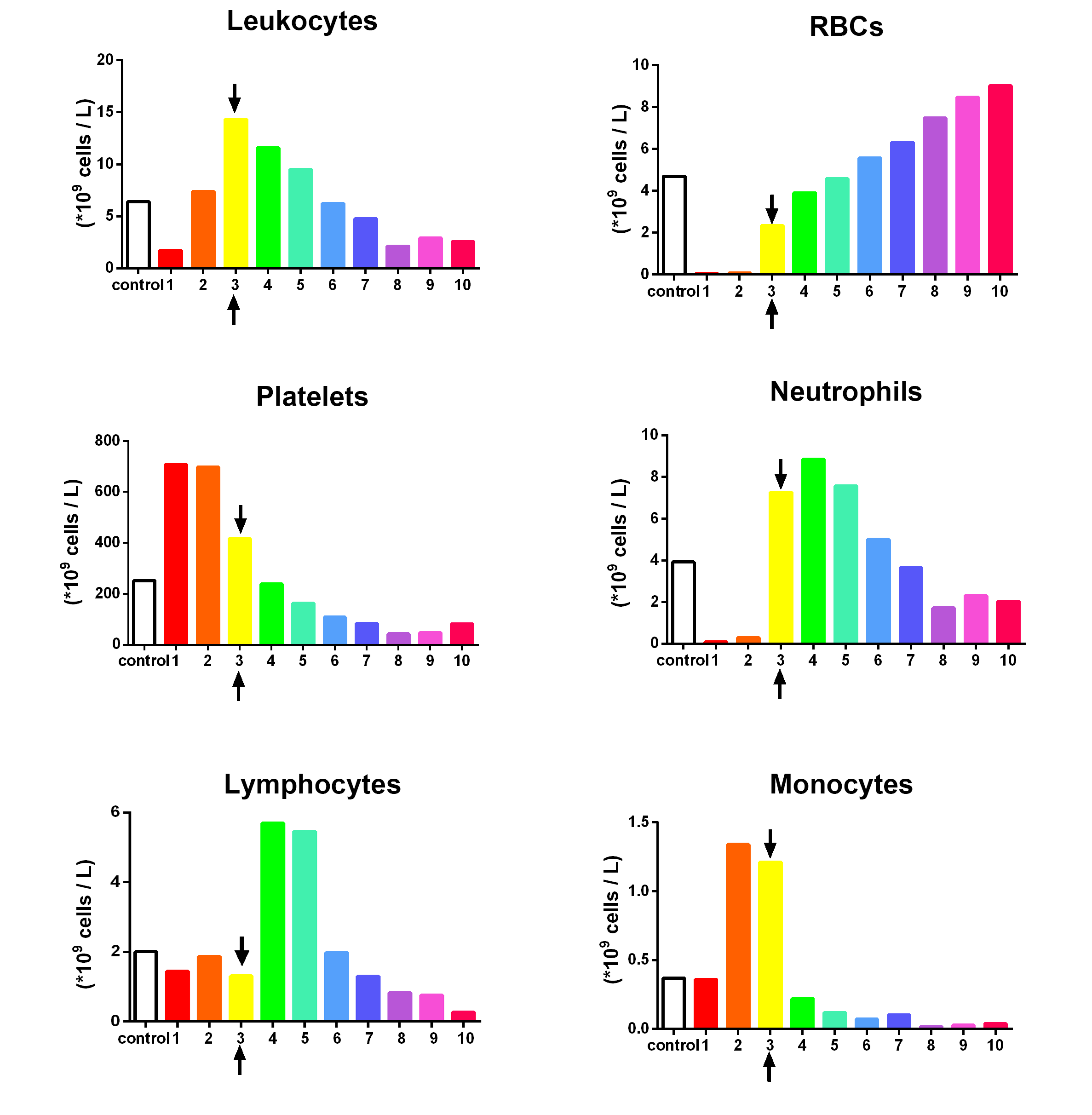

Supplement: Supplementary file 1 — Additional file 1. Variability of cell layer separation following the same protocol in 3 different individuals. One interesting observation in the present study was the variability observed among various donors when centrifugation took place at identical protocols. Note the difference in cell layer separation among 3 patients centrifuged at 700g for 5 min. While in donor one, the plasma layer separation occurred in layer 3 (Figure S1), donor 3 demonstrated separation in layer 5 (Figure S2) and demonstrated a greater than 50% increase in the total amount of plasma following identical protocols. The majority of donor samples tended to show separation at layer 4, as depicted in Figure S3. (Arrows represent the separation between the plasma and red blood cell layer (buffy coat)). [file 12903_2020_1299_MOESM1_ESM.zip › 12903_2020_1299_MOESM1_ESM/new supp. Fig.1R3.tif]

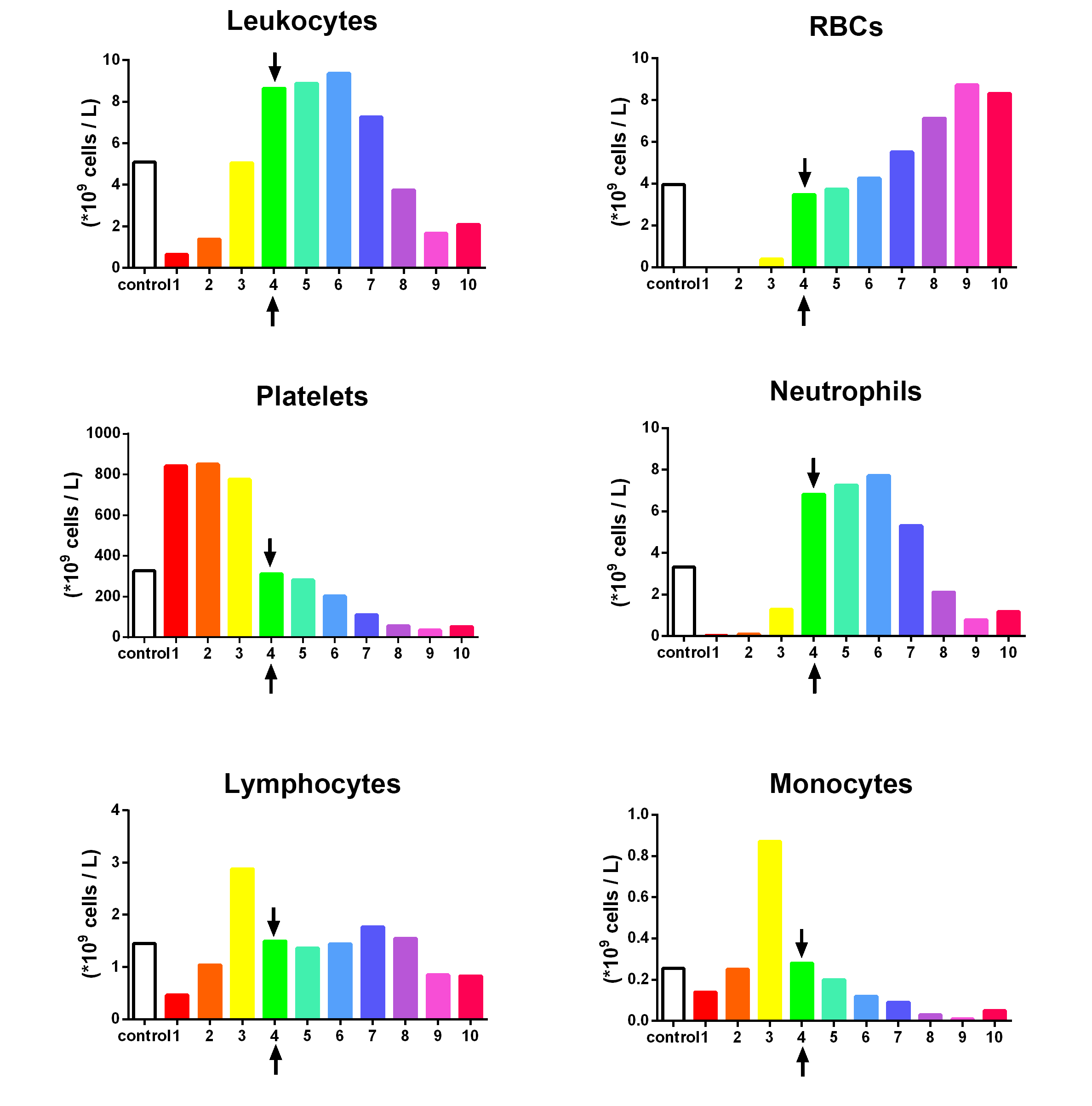

Supplement: Supplementary file 1 — Additional file 1. Variability of cell layer separation following the same protocol in 3 different individuals. One interesting observation in the present study was the variability observed among various donors when centrifugation took place at identical protocols. Note the difference in cell layer separation among 3 patients centrifuged at 700g for 5 min. While in donor one, the plasma layer separation occurred in layer 3 (Figure S1), donor 3 demonstrated separation in layer 5 (Figure S2) and demonstrated a greater than 50% increase in the total amount of plasma following identical protocols. The majority of donor samples tended to show separation at layer 4, as depicted in Figure S3. (Arrows represent the separation between the plasma and red blood cell layer (buffy coat)). [file 12903_2020_1299_MOESM1_ESM.zip › 12903_2020_1299_MOESM1_ESM/new supp. Fig.2R3.tif]

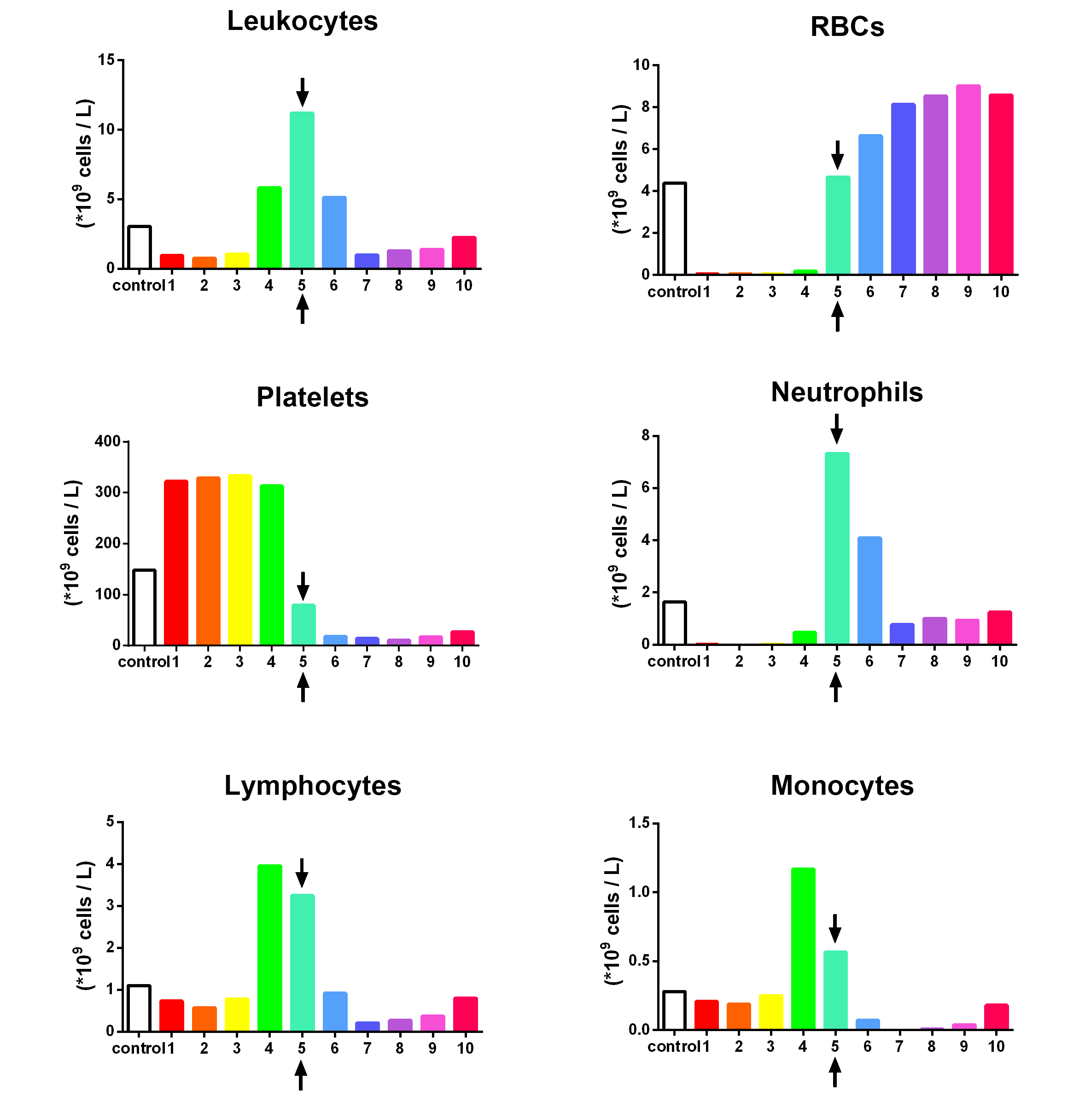

Supplement: Supplementary file 1 — Additional file 1. Variability of cell layer separation following the same protocol in 3 different individuals. One interesting observation in the present study was the variability observed among various donors when centrifugation took place at identical protocols. Note the difference in cell layer separation among 3 patients centrifuged at 700g for 5 min. While in donor one, the plasma layer separation occurred in layer 3 (Figure S1), donor 3 demonstrated separation in layer 5 (Figure S2) and demonstrated a greater than 50% increase in the total amount of plasma following identical protocols. The majority of donor samples tended to show separation at layer 4, as depicted in Figure S3. (Arrows represent the separation between the plasma and red blood cell layer (buffy coat)). [file 12903_2020_1299_MOESM1_ESM.zip › 12903_2020_1299_MOESM1_ESM/new supp. Fig.3R3.tif]
